# Problems Associated with the Microchip Data of Stray Dogs and Cats Entering RSPCA Queensland Shelters

**DOI:** 10.3390/ani5020332

**Published:** 2015-05-13

**Authors:** Emily Lancaster, Jacquie Rand, Sheila Collecott, Mandy Paterson

**Affiliations:** 1 School of Veterinary Science, The University of Queensland, Gatton, QLD 4343, Australia; E-Mail: emily.lancaster1@uqconnect.edu.au; 2 Royal Society for the Prevention of Cruelty to Animals (RSPCA), Wacol Animal Care Campus, QLD 4076, Australia; E-Mails: scollecott@rspcaqld.org.au (S.C.); mpaterson@rspcaqld.org.au (M.P.)

**Keywords:** dog, cat, microchip, data, stray, shelter, RSPCA

## Abstract

**Simple Summary:**

Microchip identification has become an important tool to reunite stray dogs and cats with their owners, and is now compulsory in most states of Australia. Improvement of the microchipping system in Australia is limited by a lack of published Australian data documenting the problems experienced by shelter staff when using microchip data to contact the owner of a stray animal. In this study we determine the character and frequency of inaccurate microchip data to identify weaknesses in the current microchipping system. This information could be used to develop strategies that increase the accuracy of microchip data that will increase the reclaiming of stray animals.

**Abstract:**

A lack of published information documenting problems with the microchip data for the reclaiming of stray animals entering Australian shelters limits improvement of the current microchipping system. A retrospective study analysing admission data for stray, adult dogs (*n* = 7258) and cats (*n* = 6950) entering the Royal Society for the Prevention of Cruelty to Animals (RSPCA) Queensland between January 2012 and December 2013 was undertaken to determine the character and frequency of microchip data problems and their impact on outcome for the animal. Only 28% of dogs and 9% of cats were microchipped, and a substantial proportion (37%) had problems with their data, including being registered to a previous owner or organisation (47%), all phone numbers incorrect/disconnected (29%), and the microchip not registered (14%). A higher proportion of owners could be contacted when the microchip had no problems, compared to those with problems (dogs, 93% *vs.* 70%; cats, 75% *vs.* 41%). The proportion of animals reclaimed declined significantly between microchipped animals with no data problems, microchipped animals with data problems and non-microchipped animals—87%, 69%, and 37%, respectively, for dogs and 61%, 33%, and 5%, respectively, for cats. Strategies are needed to increase the accuracy of microchip data to facilitate the reclaiming of stray dogs and cats.

## 1. Introduction

There are an estimated 4.2 million dogs and 3.3 million cats in Australia, with approximately 39% of households owning a dog and 29% a cat [1]. According to a 2010 survey of 1015 pet-owning households in the United States of America (USA), approximately 15% of dog and cat owners lost their pet at least once during a five-year period [2]. Stray animals are the most common source of admission to animal shelters and council pounds (municipal animal control facilities) across Australia, and euthanasia is a frequent outcome for these animals, especially cats [3,4,5,6,7]. In 2012–2013, 36% of dogs and only 5% cats entering shelters run by the Royal Society for the Prevention of Cruelty to Animals (RSPCA) in Australia were reclaimed and the euthanasia rate was 22% for dogs and 40% of cats [7].

Microchips have become an important form of identification facilitating reunification of stray dogs and cats with their owners [8]. In most states in Australia, it is now compulsory for dogs and cats to be microchipped and registered in an approved database [9]. Several studies in Australia and the USA have documented that the majority of people believe that microchipping, or at least some form of identification is important and should be compulsory for all dogs and cats [10,11,12,13].

A study of 7704 microchipped animals entering 53 shelters across the USA found that the reclaim rates for microchipped animals were substantially higher than the overall reclaim rates for the shelters—52% *versus* 22% for dogs, and 39% *versus* 2% for cats [8]. However, the study identified several key issues resulting in failure of shelters to contact the animals’ owners, including an incorrect or disconnected phone number linked to the microchip (35%), failure of the owner to respond to telephone calls or written correspondence (24%), the microchip was registered to a different group (17%) or owner (13%), or the microchip was not registered on a database (10%) [8].

Microchips with accurate contact details ensure that the owner of a lost or stray pet can be found quickly, decreasing the time required for the animal to remain with authorities, and reducing the number of animals euthanased due to insufficient space [4,8]. It also reduces the shelter staff time involved in locating owners, and therefore, shelter costs per animal. Currently, there are no published data documenting the frequency of problems experienced by animal shelter staff in Australia using microchip data to contact the owner of a stray animal.

The aim of this study was to analyse admission data for stray dogs and cats entering RSPCA-Queensland (QLD) shelters to determine the character and frequency of inaccurate microchip data used for locating owners of stray dogs and cats. This knowledge could provide a foundation for the development of evidence-based strategies to increase the effectiveness of microchips for reuniting lost pets with their owners.

## 2. Experimental Section

### 2.1. Study Design Overview and Data Collection

A retrospective single cohort study of all dog and cat admissions into RSPCA-QLD shelters from 1 January 2012 to 31 December 2013 was undertaken. Data were imported from the RSPCA’s data management program ShelterMate^©^ into Microsoft Excel for manipulation. Data obtained included animal identification number, name of shelter, date of admission, admission source, sex, breed, desex status (yes/no/unknown), date desexed, feral status (yes/no/unknown) (for cats only), age category (adult ≥16 weeks; puppy/kitten <16 weeks), desexed tattoo, VIP tag (RSPCA identification tag), council registration, primary microchip number, primary microchip implant date, secondary microchip number, secondary microchip implant date, outcome, outcome date, ID trace history, and general/ownership notes. To clarify details about shelter operations (such as data recording procedures), informal interviews were conducted with shelter management staff.

Only data for animals that met the following inclusion criteria were included in the subsequent analysis:

1. Adult dogs and cats (as classified according to RSPCA criteria) admitted as stray animals from the general public (someone claiming not to be the owner); and
2. Adult dogs and cats admitted via RSPCA animal ambulance officers after being reported as sick/injured or orphaned (included free-roaming owned, stray, feral (cats) and lost animals).

For animals admitted on >1 occasion during the study period, only data for their first admission was included in the analysis. Puppies and kittens (classified by the RSPCA as <16 weeks) were not included in the study because they are not legally required to be microchipped until 12 weeks of age [14]. Animals meeting the following criteria were excluded from the analysis:

1. Animals admitted as a “stray” by a member of the public who was subsequently found to be the owner or the alternate contact on the microchip; and
2. Dogs and cats classified as feral (*i.e.*, they displayed feral behaviour) were excluded from analyses and only the percentage microchipped reported, because a previous study identified them as largely unowned, and 95% were euthanased [3].

Animals with “yes” for desexed and no desex date listed were identified as desexed prior to admission; and all others were assumed to have not been desexed prior to admission. To determine if an animal was microchipped, the following columns from the ShelterMate^©^ data were assessed: Primary microchip number, primary microchip implant date, secondary microchip number, secondary microchip implant date, admission date and comments. Animals were deemed to be microchipped if a microchip number was listed in one of the columns and the implant date was prior to the animal’s admission to the shelter. For those animals with a microchip number listed but no implant date provided, the microchip number was manually checked against a master list of RSPCA implanted microchips to determine if it had been implanted by an RSPCA shelter, and if so, whether it was implanted prior to arrival. The total sample included 15,122 animals (7669 dogs and 7453 cats). Based on the inclusion and exclusion criteria, 2610 microchipped animals (2011 dogs and 599 cats) and 11,598 non-microchipped animals (5247 dogs and 6351 cats) were included in the analysis.

Outcomes were defined as adopted (rehomed), euthanased (humanely killed), reclaimed (by the current owner or a previous owner who was listed as the current owner on the microchip (*i.e.*, someone who owned the animal before it was sold or given to the current owner) or other. “Other” referred to animals that were listed as dead on arrival, escaped, euthanased in the field, released or transferred to another agency, stolen, unassisted death (in shelter or foster), or had an unresolved file or no outcome listed.

Five topic categories were developed to identify problems with the microchip data, collectively referred to as “microchip data problems”, in response to the information provided in the comments section for each animal. These included: (1) microchip not registered; (2) microchip registered to a previous owner or organisation; (3) microchip registered to wrong animal; (4) all phone numbers incorrect or disconnected; and (5) no answer on any phone number and unable to a leave message on any phone number, or only able to leave a message on a phone number not confirmed to be the owner’s. Animals were classified as having no problems if no issues were identified in the comments section, or if a message was able to be left on at least one phone number that was confirmed to be the owner’s (either via the message bank or the owner contacted the RSPCA and confirmed the phone number was current).

### 2.2. Statistical Analysis

Chi-square analysis was used to evaluate bivariate relationships between presence of microchip on admission and the following variables—Source (stray/ambulance), sex (male/female) and desexed status on arrival (desexed/not desexed). Animals with missing data were excluded from relevant analyses. Two logistic regression analyses, one for dogs and one for cats, were then used to examine the effect of these three variables on whether the animals were microchipped.

Two logistic regression analyses were conducted (one for dogs and one for cats) to examine the effect of microchip status (*i.e.*, non-microchipped, microchipped with no data problems identified, or microchipped with data problems identified) on reclaim status (*i.e.*, whether the animal was reclaimed or not). Animals with the outcome defined as “other” were excluded from these analyses because it could not be accurately determined if the animal or body was collected by the owner, and therefore could not be classified as either reclaimed or not. Two logistic regression analyses were conducted (one for dogs and one for cats) to examine the effect of microchip status on the proportion of animals adopted or euthanased in the non-reclaimed group. Animals with the outcome defined as “other” were also excluded from these analyses.

For reclaimed animals, Kruskal-Wallis tests were used to examine if the number of nights until the animal was reclaimed was statistically significant for animals with different microchip statuses, and statistically significant results were followed up with Mann-Whitney tests for pairwise comparison. Median and IQR are reported and the non-parametric Kruskal-Wallis tests were used because the distribution of nights until reclaimed was strongly skewed to the right.

Odds ratios (OR) and 95% confidence intervals (CI) are reported for relevant analyses, and were performed using IBM SPSS Statistics for Windows (version 21.0, 2012, IBM Corporation, Armonk, NY, USA) [15]. For all analyses, values of *p* < 0.05 were considered statistically significant.

## 3. Results

### 3.1. Microchipping Rates

Descriptive statistics of source, sex, desexed status, microchip status, and outcome of the dogs and cats included in the study are shown in Table 1. Overall microchipping rates were low—only 28% of incoming dogs and 9% of incoming cats were categorised as having microchips prior to admission. Results of Chi-square analysis examining the bivariate relationship between source, sex and desexed status on whether a dog or cat was microchipped on admission are shown in Table 2. These results showed that source (stray/ambulance) was not statistically significant (*p* = 0.6) with whether dogs were microchipped on arrival, but it was for cats (*p* ≤ 0.001); sex was not statistically significant for dogs (*p* = 0.1) but it was for cats (*p* = 0.009) with a lower proportion of females microchipped, and desexed status was statistically significant for dogs (*p* ≤ 0.001) and cats (*p* ≤ 0.001). Table 3 shows the results from logistic regression analyses that examine the effect of source, sex and desexed status on the likelihood that a dog or cat would be microchipped on admission. In this multivariate analysis, cats were significantly (*p* = 0.002) more likely to be microchipped if they were admitted via ambulance (OR 1.4); dogs were marginally less likely to be microchipped if they were female (OR 0.9, *p* = 0.03, same odds for cats but not statistically significant); and dogs and cats were significantly (*p* ≤ 0.001) more likely to be microchipped if they were desexed (dogs: OR 3.3; cats: OR 12.7).

**Table 1:** Descriptive statistics of stray adult dogs and cats admitted to RSPCA-QLD shelters (January 2012–December 2013). Percentage of source, sex, desexed status, microchip status and outcome is shown in brackets.

Total	Total (%)		Dogs (%)		Cats (%)	
	14208		7258		6950	
**Source**						
Stray	11,887	(84)	6283	(87)	5604	(81)
Ambulance	2321	(16)	975	(13)	1346	(19)
**Sex**						
Male	7269	(51)	3859	(53)	3410	(49)
Female	6704	(47)	3370	(46)	3334	(48)
Unknown	235	(2)	29	(<1)	206	(3)
**Desexed status**						
No	8952	(63)	4443	(61)	4509	(65)
Yes	3916	(28)	2054	(28)	1862	(27)
Unknown	1340	(9)	761	(10)	579	(8)
**Microchip status**						
Non-microchipped	11,598	(82)	5247	(72)	6351	(91)
Microchipped	2610	(18)	2011	(28)	599	(9)
**Outcome**						
Adopted	6159	(43)	2159	(30)	4000	(58)
Euthanased	2903	(20)	1167	(16)	1736	(25)
Reclaimed	3783	(27)	3229	(44)	554	(8)
Other	1363	(10)	703	(10)	660	(9)

**Table 2:** Cross-tabulation of source, sex and desexed status for non-microchipped and microchipped stray adult dogs and cats admitted to RSPCA-QLD shelters (January 2012–December 2013). Percentage of non-microchipped and microchipped animals for source, sex and desexed status is shown in brackets.

Admission characteristics	Dogs						Cats					
	Total	Not Microchipped (%)		Microchipped (%)		*p*-value	Total	Not Microchipped (%)		Microchipped (%)		*p*-value
**Source**												
Stray (*n* = 11,887)	6283	4535	(72)	1748	(28)		5604	5164	(92)	440	(8)	
Ambulance (*n* = 2321)	975	712	(73)	263	(27)	0.583	1346	1187	(88)	159	(12)	0.001
**Sex** ^b^												
Male (*n* = 7193)	3859	2762	(72)	1097	(28)		3334	3016	(91)	318	(10)	
Female (*n* = 6780)	3370	2465	(73)	905	(27)	0.136	3410	3146	(92)	264	(8)	0.009
**Desexed status** ^c^												
No (*n* = 8952)	4443	3570	(80)	873	(20)		4509	4401	(98)	108	(2)	
Yes (*n* = 3916)	2054	1146	(56)	908	(44)	0.001	1862	1405	(76)	457	(25)	0.001

^a^ *p*-value is for chi-square analysis assessing if there is a statistically significant association between source, sex and desexed statistic and being microchipped on admission; ^b^ Animals of known sex; ^c^ Animals of known desexed status.

**Table 3:** Results of a multivariate logistic regression model describing the odds ratios for presence of a microchip in stray adult dogs and cats admitted to RSPCA-QLD shelters (January 2012–December 2013).

Admission characteristics	Dogs		Cats	
	OR (95% CI)	*p*-value	OR (95% CI)	*p*-value
**Source**				
Stray	Reference		Reference	
Ambulance	0.9 (0.8–1.1)	0.365	1.4 (1.1–1.8)	0.002
**Sex** ^a^				
Male	Reference		Reference	
Female	0.9 (0.8–1.0)	0.027	0.9 (0.8–1.1)	0.286
**Desexed status** ^b^				
No	Reference		Reference	
Yes	3.3 (2.9–3.7)	<0.001	12.7 (10.2–15.9)	<0.001

^a^ Animals of known sex; ^b^ Animals of known desexed status; CI, confidence interval; OR, odds ratio.

### 3.2. Reclaim Rates and Owners Contacted

Reclaim rates were significantly (*p* ≤ 0.001) higher for animals with microchips than without—80% *versus* 37% for dogs (OR 7.0 (95% CI: 6.2 to 8.0)), and 51% *versus* 5% for cats (OR 20.2 (95% CI: 16.4 to 25.0)). Of all incoming microchipped animals (excluding those with an outcome of “other”), 80% of 2439 current owners could be contacted (85% of 1892 dogs; 62% of 547 cats). Some dog (6%; *n* = 113) and cat (14%; *n* = 74) owners that were successfully contacted did not want to reclaim their animal. Three microchipped dogs that had been involved in attacks on humans and/or a pet owned by another member of the public prior to admission were prohibited from being reclaimed by the council (and were subsequently euthanased).

### 3.3. Microchip Data Problems

No problems were identified with the microchip data for 63% of dogs and 63% of cats that were microchipped (Table 4). A significantly (*p* ≤ 0.001) higher proportion of dog and cat owners (current owners) could be contacted in the group with no microchip data problems compared to the group with problems—93% *versus* 70% for dogs (OR 6.1 (95% CI: 4.5 to 8.1)) and 75% *versus* 41% for cats (OR 4.2 (95% CI: 2.9 to 6.2)). For animals with microchip data problems, owners were successfully contacted using other methods (for example, using information linked to the animal’s council registration tag or owner tag) or the owner contacted the RSPCA themselves. Of the animals with no microchip data problems, some owners were unable to be contacted because they failed to return phone calls or respond to correspondence from the RSPCA.

Importantly, the odds of dogs and cats being reclaimed decreased significantly (*p* ≤ 0.001) between the different microchip status groups (Table 5). Animals with no microchip data problems had 11.5 (dogs) and 31.2 (cats) higher odds of being reclaimed than non-microchipped animals. However, dogs and cats with microchip data problems were only 3.8 (dogs) and 9.7 (cats) times more likely to be reclaimed than non-microchipped animals. Of the animals that were not reclaimed (*i.e.*, were adopted or euthanased), there was no impact on euthanasia between the different microchip statuses for dogs (Table 6). Cats with no microchip data problems had lower odds of being euthanased (OR 0.6) than cats with no microchip. Conversely, cats with microchip data problems were at higher odds of euthanasia (OR 1.5) than cats with no microchip, although the numbers of cats with microchips were low.

The main problems associated with microchip data were the microchip was linked to a previous owner or organisation (47%), all telephone numbers were incorrect or disconnected (29%), and no contact details were linked to the microchip or the microchip was not registered with a database company (14%). Of those registered to a previous owner or organisation, the microchip was linked to one of the following: Previous owner (72% of dogs and 81% of cats), breeder (20% of dogs and 7% of cats), pet shop (5% of dogs and 9% of cats), rescue group or shelter (3% of dogs and 2% of cats) (rescue *n* = 9, shelter *n* = 2) and veterinarian (1% of cats).

**Table 4:** Frequency of each problem identified with the microchip data of 2439 microchipped stray adult dogs and cats entering RSPCA-QLD shelters (January 2012–December 2013). Percentages of the problems identified in each category are shown in brackets.

Microchip data problems	Total ^a^ (%) (*n* = 2,439)		Dogs ^a^ (%) (*n* = 1,892)		Cats ^a^ (%) (*n* = 547)	
**Presence of problems**						
No problems	1539	(63)	1196	(63)	343	(63)
Problems identified	900	(37)	696	(37)	204	(37)
**Specific problems identified**						
Microchip registered to previous owner or organisation	421	(47)	333	(48)	88	(43)
Microchip not registered	124	(14)	101	(15)	23	(11)
Microchip registered to different animal	3	(<1)	2	(<1)	1	(<1)
All numbers incorrect/disconnected	261	(29)	194	(28)	67	(33)
No answer on any phone number & (1) unable to leave messages; or (2) able to leave message only on an unconfirmed number	90	(10)	65	(9)	25	(12)

^a^ Animals whose outcome was either adopted, euthanased or reclaimed.

**Table 5:** Results of logistic regression analyses describing the relationship between microchip status and reclaim outcome of stray adult dogs and cats admitted to RSPCA-QLD shelters (January 2012–December 2013). Percentages for reclaim status within each microchip status are shown in brackets.

Microchip status	Total	Not Reclaimed ^a^ (%)		Reclaimed (%)		OR (95% CI)	*p*-value
**Dogs**	6555	3326	(51)	3229	(49)		
Non-microchipped	4663	2952	(63)	1711	(37)	Reference	
Microchipped and No problems	1196	156	(13)	1040	(87)	11.5 (9.6–13.8)	<0.001
Microchipped and Problems	696	218	(31)	478	(69)	3.8 (3.2–4.5)	<0.001
**Cats**	6290	5736	(91)	554	(9)		
Non-microchipped	5743	5466	(95)	277	(5)	Reference	
Microchipped and No problems	343	133	(39)	210	(61)	31.2 (24.3–39.9)	<0.001
Microchipped and Problems	204	137	(67)	67	(33)	9.7 (7.0–13.2)	<0.001

^a^ Adopted and euthanased animals collectively; CI, confidence interval; OR, odds ratio.

**Table 6:** Results of logistic regression analyses describing the relationship between microchip status and euthanasia of non-reclaimed (*i.e.*, adopted or euthanased) stray adult dogs and cats admitted to RSPCA-QLD shelters (January 2012–December 2013). Percentages for adopted or euthanased status within each microchip status are shown in brackets.

Microchip status	Total	Adopted (%)		Euthanased (%)	OR (95% CI)		*p*-value
**Dogs**	3326	2159	(65)	1167	(35)		
Non-microchipped	2952	1913	(65)	1039	(35)	Reference	
Microchipped and No problems	156	104	(67)	52	(33)	0.9 (0.7–1.3)	0.635
Microchipped and Problems	218	142	(65)	76	(35)	1.0 (0.7–1.3)	0.921
**Cats**	5736	4000	(70)	1736	(30)		
Non-microchipped	5466	3813	(70)	1653	(30)	Reference	
Microchipped and No problems	133	105	(79)	28	(21)	0.6 (0.4–0.9)	0.024
Microchipped and Problems	137	82	(60)	55	(40)	1.5 (1.1–2.2)	0.014

CI, confidence interval; OR, odds ratio.

Of the dogs and cats whose owner was unable to be contacted due to incorrect/disconnected phone numbers, only 13% (33/259) of dogs and 11% (10/92) of cats had an alternate contact that was able to provide the owner’s current details, or who contacted the owner listed on the microchip on behalf of the RSPCA. Of animals whose microchip was linked to a previous owner or organisation, only 36% (153/421) could provide the RSPCA with current details of the owner (46% could not provide details, and for 18%, it was unknown if they could provide current owner details). For a small number of dogs (8%; 27/333) and cats (9%; 8/88) where the microchip was linked to a previous owner, and the current owner could not be contacted or did not want to reclaim the animal, the owner listed on the microchip reclaimed the animal.

Two microchips were present in 0.92% of microchipped animals (24/2,610; 16 dogs and 8 cats). There were 3 animals (2 dogs and 1 cat) whose microchip was registered to the wrong animal. Two dogs (0.08% of total microchipped admissions) had a microchip that was considered to have migrated as their microchips were detected in a location other than the standard microchip implantation sites developed by the World Small Animal Veterinary Association (WSAVA) Microchip Subcommittee [16].

### 3.4. Nights Taken to Reclaim Animals

Reclaimed dogs and cats with no microchip data problems spent significantly (*p* ≤ 0.001) less time in the shelter than those without microchips (median 0 *versus* 1 night for dogs; median 0 *versus* 2 nights for cats); but there was no difference in time between animals with microchip data problems and those with no microchip (dogs: *p* = 0.7; cats: *p* = 0.8) (Table 7).

The owners identified on the microchip of 8 dogs and 1 cat claimed that the animal had been stolen from 4 weeks to 4 years previously, and 4 of these dogs and the cat were reclaimed by the owner listed on the microchip. The owners of 13 animals (3 dogs and 10 cats) reported that the animal had been missing for a period longer than 2 months, and all were reclaimed by the owner on the microchip. Of these, 1 dog and 1 cat had been recorded in the file as missing for “over a year”, 1 cat had been missing for 3 years and 1 cat had been missing for 6 years. One dog had an international microchip.

**Table 7:** Number of nights spent at shelter before reclaim for stray adult dogs and cats admitted to RSPCA-QLD shelters (January 2012–December 2013) and Mann-Whitney pairwise comparisons of microchip status groups.

Microchip status	Dogs				Cats			
	Total ^a^	Median	IQR	*p*-value	Total ^a^	Median	IQR	*p*-value
Non-microchipped	1662	1	0–2		260	2	1–4	
Microchipped without problems	1010	0	0–1	0.001	205	0	0–2	0.001
Microchipped with problems	456	1	0–2	0.675 (comparison with non-microchipped)	64	1.5	1–4	0.820 (comparison with non-microchipped)
Microchipped with problems				0.001 (comparison with microchipped without problems)				0.001 (comparison with microchipped without problems)

^a^ Reclaimed animals with known admission and outcome dates; IQR, interquartile range.

### 3.5. Feral Animals

Four hundred and twenty-seven cats (2 microchipped) were classified as feral and excluded from statistical analyses. Eleven dogs (5 microchipped) had a feral status recorded, a meaningless classification for dogs and represented a recording error.

## 4. Discussion

The proportion of microchipped dogs (28%) and cats (9%) entering RSPCA-QLD shelters was considerably lower than the general microchip status in the community reported in a 2013 survey of 1734 Australian households, which found 76% of owned dogs and 64% of owned cats were microchipped [1]. From July 2009, it is a legal requirement that all animals in QLD be microchipped before 12 weeks of age and prior to sale or transfer [14]. This suggests that a large proportion of animals entering shelters either come from owners who are less diligent about pet care and ownership responsibilities, or for cats, come from the feral or semi-owned populations rather than the owned pet population [3,4,5,17]. Of owner-surrendered dogs and cats entering shelters in Australia, only 41% of dogs and 8%–34% of cats are desexed [3,4,5,6]. As 78% of owned dogs and 91% of owned cats are reported to be desexed [1], this suggests that owners who surrender animals to shelters are less diligent about having their pet desexed, and it is possible that owners who let their pet stray are also less diligent about other aspects of pet care such as registration and microchipping.

Studies in Australia and the USA have found common reasons for not microchipping include expense and difficulty due to the required veterinary visit [12,18,19]. Microchipping a pet costs $AUD 30–50 plus the veterinary consultation fee [20]. Councils and welfare organisations often have reduced-cost microchipping days; however, cat owners are less likely to take advantage of this for fear that their pet will escape [21], so further research is required to identify methods to combat barriers to microchipping, particularly of cats. Many owners also believe that indoor-only cats do not require a microchip, but a survey of owners of lost pets in the USA found 41% of lost cats were classified as indoor only [18,19,22].

The reclaim rate was significantly higher for animals with microchips (80% *versus* 37% for dogs and 51% *versus* 5% for cats), supporting microchips as an important form of identification. However, problems with microchip data reduced overall effectiveness in being able to reunite lost animals with owners. In the USA study of 7704 microchipped animals entering animal shelters, issues with microchip data meant that the shelter was unable to contact the owners of 26% of dogs and 37% of cats admitted to the shelters [8]. In our study, the proportion of dog owners that could not be contacted was lower (15%), but was almost equal for cats (38%) to this USA study.

A higher percentage of owners could be contacted in the group with no microchip data problems compared to the group with problems (93% *versus* 70% for dogs and 75% *versus* 41% for cats). The number of nights taken to reclaim an animal with microchip data problems was also significantly longer than those without problems (1 *versus* 0 nights for dogs; 1.5 *versus* 0 nights for cats), and was not significantly different to those animals that had no microchip. This highlights that accurate contact details can significantly reduce the time for reunification of lost pets, thereby reducing the resources required by shelters to house these animals and also, reduces the reclaim fee charged to the owner (due to the sustenance fees charged). The short median time for reclaim of microchipped and non-microchipped animals likely reflects that people who reclaim their pet are often actively searching for the animal at the time it is admitted to the shelter. It also suggests that the probability of being reclaimed decreases the longer an animal is in the shelter.

We did not determine whether animals in this study had visual identification. However, the higher proportion of microchipped dog owners than cat owners (and faster reclaim rate of dogs) may be because dogs are more likely than cats to also have visual identification (either council or owner’s tag), providing an alternative source of owner contact information [2,4,5,6,10,13,22,23,24]. Dog owners are also more likely to call or visit an animal care agency (and within a shorter period of time) than are cat owners [2,22,23]. RSPCA-QLD shelters maintain an internal database of all animals entering and leaving their shelters and manage a lost and found website that can be checked for owner details. It is likely that these methods assist shelters to contact owners when they could not use the microchip data.

Of the microchipped animals whose owners were successfully contacted, a higher proportion of dog owners than cat owners wanted to reclaim their animal—94% *versus* 86%. A similar situation was seen in the USA study where, of the microchipped animals whose owners were successfully contacted (74% of dog and 64% of cat owners) a higher proportion of dog owners (76%) compared to cat owners (61%) wanted to reclaim their animal.

The most common problem identified with the microchip data was that it was registered to a previous owner or organisation (48% of dogs and 43% of cats). This issue was also highlighted in the USA study, where, of the animals whose owner could not be found, 17% were registered to a previous organisation and 13% were registered to a previous owner [8]. This issue increases the time and resources required by RSPCA-QLD to find the current owner (as is evidenced by the fact that only 36% were able to provide the current details for the owner), and questions the legality of true ownership of the animal. This is a significant issue in Australia because breeders and friends or neighbours are two of the most common sources of animals—30% and 20% respectively for dogs, and 14% and 22% respectively for cats [1]. Currently, there is no legal requirement for previous owners to transfer ownership details on the microchip to new owners [14]. Successful transfer requires appropriately signed forms be sent to the registry, and many people may mistakenly believe that the previous owner has done this. Given the high proportion of microchips registered to a previous owner or organisation, strategies are needed to facilitate ease of ownership transfer. It may be more appropriate for a process similar to car registration transfer in Queensland to be implemented, where it is a legal requirement that the seller provides the buyer with the paperwork needed for ownership transfer. The details of both parties are on the registration transfer application and both parties keep a copy. In the event that the purchaser does not lodge the transfer documents, the seller may lodge their section of the form to remove any accountability for any offenses committed by the new owner [25].

Very few animals were incorrectly registered to a shelter, despite 15% of dogs and 22% of cats in Australia being acquired from the RSPCA or another animal shelter [1]. This represents a considerable investment of resources by the RSPCA and other shelters to change ownership information on the microchip database at the time of adoption, given the large number of animals being adopted. Under Queensland law, it is the responsibility of the new owner and not the shelter to update the microchip database [14]. All phone numbers linked to the microchip were incorrect or disconnected in 28% of dogs and 33% of cats, and for a further 9% of dogs and 12% of cats, no message could be left on the phone, or the phone number could not be confirmed as correct. Similarly, in the USA study, of owners who were unable to be contacted, for 35% this was due to incorrect/disconnected phone numbers being linked to the microchip [8]. In our study, very few (13% of dogs and 11% of cats) were noted in their comments section to have an alternate contact that was able to provide details of the owner.

Our study highlights the need for strategies to ensure owners maintain accurate contact details on the microchip, even for animals that were believed to be stolen and/or have been missing for long periods of time. Owners should be encouraged to provide an alternate contact in the event that contact cannot be established with the owner on their listed phone numbers. Accuracy of microchip data may be increased through appropriate education campaigns targeted at pet owners encouraging them to maintain up-to-date details, and through implementation of a Check-the-Chip-Day [26] (as has been implemented in the USA), which may increase levels of compliance. Veterinarians could also assist in increasing accuracy of microchip data. A survey of members of the Society of Practicing Veterinary Surgeons in the United Kingdom in 2013 found that 83% of the 286 respondents would be prepared to routinely scan dogs on first presentation to their surgery, and 68% would be prepared to compare the owner’s details to that held on the database [27]. Strategies such as emails or text messages from microchip database companies reminding owners to update their pets’ details could increase the accuracy of the data held, and has recently been implemented by at least one microchip company in Australia using email (per comm Rick Walduck, Central Animal Records). Further research is needed to determine why owners fail to update their contact details, so this issue can be addressed.

Of the animals with microchips that had data problems, a substantial proportion of microchips were not registered (15% of dogs and 11% of cats (5% of total admissions, 124/2,439)). These results are lower than that found in the USA study—Of the animals whose microchip registration was checked, 42% (814/1,943) were not registered in a microchip registry [8]. However, at the time of the USA study, it was not the legal responsibility of the implanter to register the microchip with a microchip registry, and was often left up to the animal’s owner [8]. Under the *QLD Animal Management (Cats and Dogs) Act 2008*, from July 2009, the person implanting the microchip must register the microchip with a microchip registry within seven days [14].

The USA study found that of the 13% of animals whose microchip was detected at a time other than on admission to the shelter, 98% had been missed at a previous scanning [8]. At least 2 animals in our study had microchips that were not detected at first arrival, although this might be underreported, given the retrospective nature of our study. However, unlike the USA, to ensure all scanners are capable of detecting all microchips, Australia has implemented legislation requiring all microchips and scanners sold in Australia conform to those dictated by the International Standards Organisation (ISO) [28,29]. Studies have shown that many scanners used in the USA are not capable of detecting every frequency microchip sold there [30,31].

Two dogs had a microchip that was considered to have migrated. The migration rate found in our study (0.8%) is consistent with the results of previous studies—0% to 1.6% [8,18,32]. The elbow, shoulder and sternum are the most common areas that microchips migrate to [33]. This highlights the importance of appropriate implantation procedures, using an appropriate scanning technique and scanning animals more than once [8,16,28].

An additional problem faced by shelters in determining the current owner based on microchip information, is the ability of owners to register the microchip with more than one microchip registry. Shelters and owners can determine which microchip registries a microchip is registered to (and be provided with their contact details) by inputting the animal’s microchip number into the Pet Address online search engine [34]. However, staff at shelters must contact each registry the microchip is registered with, to determine which one holds current owner information, and if different owners are registered on each database, the discrepancy must be investigated before the animal can be reclaimed (per comm RSPCA). This issue increases the time and resources required by the shelter to determine who is the true owner, and must be addressed. One solution would be sharing of data between databases, and the generation of an automatic email or phone message to owners where inconsistencies are detected.

Of note, although there were statistically significant differences in reclaim rates between microchip groups, there were no differences in the percentage of dogs rehomed or euthanased between microchip groups. This suggests that other factors such as dog-related factors influence adoption and euthanasia, and that human (owner)-related factors are more important influencers of reclaim of lost dogs and of microchipping. It could be hypothesized that dog-related factors such as behavior, breed and age that influence the surrender of animals to shelters and outcome in the shelter [6,35], are less significant than owner-related factors in determining if a lost dog is reclaimed. For cats, those with no microchip data problems were at significantly lower risk of euthanasia than those with problems, which may reflect that they have been free-living for less time and potentially are healthier and/or friendlier to humans. Stray cats are common in urban areas [3,4,5,17,21], and if they have been free-living for some time and microchipped, more microchip data problems would be expected than for a recently lost cat.

For cats, microchips could be a vital tool to differentiate frightened owned or previously owned cats from feral cats which are considered pest animals [36], because both may exhibit similar “feral” behaviours on admission [3,37,38]. Most shelters classify cats as feral based on a subjective behavioural assessment on admission, and several studies of cat admissions to shelters have found this can often be an inaccurate measure of their normal behavior [3,37,38].

Microchip data enabled 25 owner-surrendered animals to be identified. However, this was after the animal was admitted as a stray, meaning the owner avoided paying the surrender fee, and the shelter spent time and resources attempting to contact an owner that did not want the animal. Some owners may be unwilling to pay the small surrender fee charged by RSPCA-QLD (per comm RSPCA), which is not requested for stray admissions, or not want to admit to surrendering their own animal, so claim it to be a stray (per comm RSPCA). This highlights a need to determine the feasibility of comparing microchip data to that of the person admitting it as a stray, prior to accepting the animal. For non-microchipped animals, the number of owners falsely admitting their animal as a stray could be much higher, and further research is needed to investigate this.

The main limitation of our study was related to correctly identifying animals that were microchipped on entry into the shelter (compared to after entry), and the interpretation of the comments section containing 3–1200 words for each animal, to identify problems associated with the microchip data. The frequency of microchip data problems identified is likely an underestimation, because methods other than the microchip might have been used to locate the owner, and a microchip problem might not have been recorded by staff. A prospective study using drop down menus for data entry might be more accurate, as used in the USA study [8]. Although data for the whole of Australia was not analysed, dog and cat admissions to the RSPCA-QLD constitute 32% of dog and 27% of cat RSPCA admissions nationally [7], so our data represents a substantial proportion of animals entering RSPCA shelters around Australia. It is possible that the level of attachment to the pet by the owner influences their likelihood of maintaining accurate contact details and/or reclaiming the animal, in the event that it has entered a shelter or council pound. Further research is required to determine if these are associated.

## 5. Conclusions

The reclaim rate of microchipped animals is significantly higher than for those without, supporting the notion that microchips are a vital form of identification. However, microchip data problems reduce the likelihood that an animal’s owner will be contacted and the animal reclaimed. Although many owners of animals with microchip data problems can be contacted using other methods, these animals spend longer at shelters before they are reclaimed. Given the high proportion of animals with microchips registered to a previous owner or organisation, strategies are needed to facilitate ease of ownership transfer. Owner education campaigns and annual reminders from microchip registries may reduce the frequency of microchips linked to out-of-date contact details (*i.e.*, incorrect and disconnected phone numbers). Based on the high proportion of microchips that were not registered, investigation is needed to determine if implanters are adhering to current QLD legislation requiring that they register the microchip. To increase the reclaim rate for cats, further research is needed to identify why dog owners (regardless of whether microchip data problems are present or not) are more likely to be contacted than cat owners, and why, of those that are contacted, dog owners are more likely to reclaim their pet.
